# Detection and Genotyping of *Coxiella burnetii* in Pigs, South Korea, 2014–2015

**DOI:** 10.3201/eid2212.161236

**Published:** 2016-12

**Authors:** Min-Goo Seo, In-Ohk Ouh, Seung-Hun Lee, Dongmi Kwak

**Affiliations:** Animal and Plant Quarantine Agency, Gimcheon, South Korea (M.-G. Seo, I.-O. Ouh, S.-H. Lee);; Kyungpook National University, Daegu, South Korea (M.-G. Seo, S.-H. Lee, D. Kwak);; Kyungpook National University Cardiovascular Research Institute, Daegu (D. Kwak)

**Keywords:** Coxiella burnetii, bacteria, pigs, humans, transmission, blood, tissue, serologic testing, phylogeny, South Korea, genotyping, detection, potential reservoir, epidemiology, prevalence, zoonoses, Q fever

## Abstract

We assessed *Coxiella burnetii* prevalence and genotypes in pigs in South Korea during 2014–2015. Prevalence was low among 1,030 samples tested by ELISA and immunofluorescent assay and 1,124 samples tested by PCR. Despite this finding, possible transmission of *C*. *burnetii* from pigs to humans cannot be excluded.

Q fever is a zoonotic disease caused by the extremely infectious bacterium *Coxiella burnetii*. Humans can be infected by inhalation of infectious aerosols or contaminated dust from infected ruminants or through contact with infected animal products. Ruminants are known as the primary reservoirs for the bacterium. Wildlife may also serve as reservoirs (*1*). However, epidemiologic data on the occurrence of *C. burnetii* in pigs are limited. Their susceptibility to *C. burnetii* infection has been confirmed by the presence of serum antibodies (*2*), but strong evidence for pigs serving as reservoirs of *C. burnetii* is lacking. In addition, transmission of *C. burnetii* from pigs to humans has not been confirmed.

In the veterinary field, commercial immunologic methods are the easiest to interpret and are used at the herd level to detect *C. burnetii* infection or exposure within a population of animals (*3*). In South Korea, there have been several studies on *C. burnetii* in ruminants (*4*,*5*), but studies evaluating *C. burnetii* in pigs are lacking. As first step toward understanding the epidemiology of *C. burnetii* in pigs, we evaluated the prevalence and genotypes of this bacterium in pigs reared in Gyeongsang Province, South Korea.

## The Study

**Figure 1:**
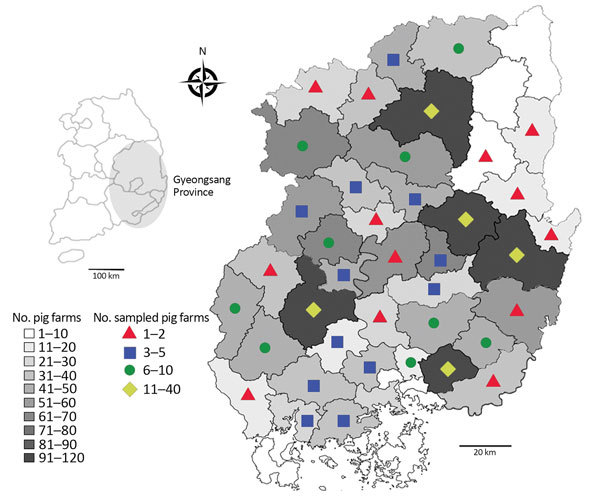
Number of pig farms in the provincial administrative districts and number of farms on which pigs were sampled for the detection and genotyping of *Coxiella burnetii*, Gyeongsang Province, South Korea, 2014–2015. The number of samplings was based on the number of pigs and farms within each of the province’s administrative districts.

During 2015 in South Korea, a total of 10,332,000 pigs were raised, of which 2,338,521 (22.6%) were raised on 1,134 farms in Gyeongsang Province (*6*). For this study, we collected 1,030 blood and 97 tissue samples from pigs (645 breeding and 479 fattening pigs) reared on 209 pig farms in Gyeongsang Province during 2014–2015. Sample size was determined using a formula with an expected disease prevalence of 50%, accepted absolute er­ror of 5%, and CI of 99% in a simple random sampling design (Technical Appendix); a minimum of 664 samples were required. Samples were collected by practicing veterinarians during treatment or regular medical checkups; ethical approval was not required. The number of samplings was based on the number of pigs and farms within each of the Province’s administrative districts (Figure 1). 

Whole blood was used for PCR; the serum was separated for serologic testing. Lung, lymph node, liver, spleen, and kidney samples were collected for differential diagnosis of diseases in pigs that aborted or had a stillbirth, respiratory symptoms, or weakness.

To detect *C. burnetii*–positive samples, we used 2 different assays and nested PCR. We used an indirect multispecies ELISA (ID Screen Q Fever Indirect Multi-species Kit; IDvet, Montpellier, France) according to the manufacturer’s instructions to detect *C. burnetii* antibodies in samples; a sample optical density to positive-control optical density value of >50% was considered positive. We also performed an indirect immunofluorescence assay (IFA), using the *Coxiella burnetii* (Q Fever) FA Substrate Slide (VMRD, Pullman, WA, USA), as recommended by the manufacturer; titers >64 to phase-1 or phase-2 antigens were considered seropositive. We used the DNeasy Blood and Tissue Kit (QIAGEN, Hilden, Germany) according to the manufacturer’s instructions to extract DNA from whole blood and tissue samples. The *Coxiella* 16S rRNA gene in extracted DNA was then amplified using nested PCR and sequencing primers (Technical Appendix). We sequenced amplification products with Macrogen (Seoul, South Korea) and analyzed results using sequence alignment programs and statistical methods (Technical Appendix).

Of the 1,030 sampled pigs, 70 (6.8%) were positive for *C. burnetii* by ELISA (Table); these pigs were from 32 (15.3%) of the 209 sampled farms. Two of the 32 farms had 8 positive pigs each; the other 30 had 1–3 positive pigs each. Fifty-three (5.2%) sampled pigs had samples identified as phase-1 or phase-2 antigen seropositive by IFA; these samples were also seropositive by ELISA. An additional 17 samples seropositive by ELISA were seronegative by IFA. *C. burnetii* seroprevalence was significantly higher (p<0.0001) in breeding than in fattening pigs by ELISA and IFA.

**Table:** Assay determinations of *Coxiella burnetii* prevalence among different types of pigs reared in Gyeongsang Province, South Korea, 2014–2015

Assay	No. positive pigs/no. total*	% Pigs positive (95% CI)
ELISA		
Breeding pigs		
Farms	**29/101**	28.7 (19.9–37.5)
Pigs	**66/637**	10.4 (8.0–12.7)
Fattening pigs		
Farms	3/108	2.8 (0–5.9)
Pigs	4/393	1.0 (0.1–2.0)
Subtotal		
Farms	32/209	15.3 (10.4–20.2)
Pigs	70/1,030	6.8 (5.3–8.3)
IFA		
Breeding pigs		
Phase-1 antigens	**46/637**	7.2 (5.2–9.2)
Phase-2 antigens	**48/637**	7.5 (5.5–9.6)
Phase-1 or -2 antigens	**49/637**	7.7 (5.6–9.8)
Fattening pigs		
Phase-1 antigens	4/393	1.0 (0–2.0)
Phase-2 antigens	4/393	1.0 (0–2.0)
Phase-1 or -2 antigens	4/393	1.0 (0.1–2.0)
Subtotal		
Phase-1 antigens	50/1,030	4.9 (3.5–6.2)
Phase-2 antigens	52/1,030	5.0 (3.7–6.4)
Phase-1 or -2 antigens	53/1,030	5.2 (3.8–6.5)
PCR		
Breed type		
Breeding pigs	3/645	0.5 (0–1.0)
Fattening pigs	0/479	0
Sample type		
Blood	2/1,030	0.2 (0–0.5)
Tissue	1/94	1.1 (0–3.1)
Subtotal	3/1,124	0.3 (0–0.6)

*Bold indicates significantly different (p<0.05) compared with fattening pigs.

ELISA and IFA results were in agreement for 1,013 (98.4%) of the 1,030 samples; 53 (5.2%) samples were positive, and 960 (93.2%) were negative. The Cohen κ coefficient was 0.85 (i.e., very good agreement; 95% CI 0.79–0.92).

**Figure 2:**
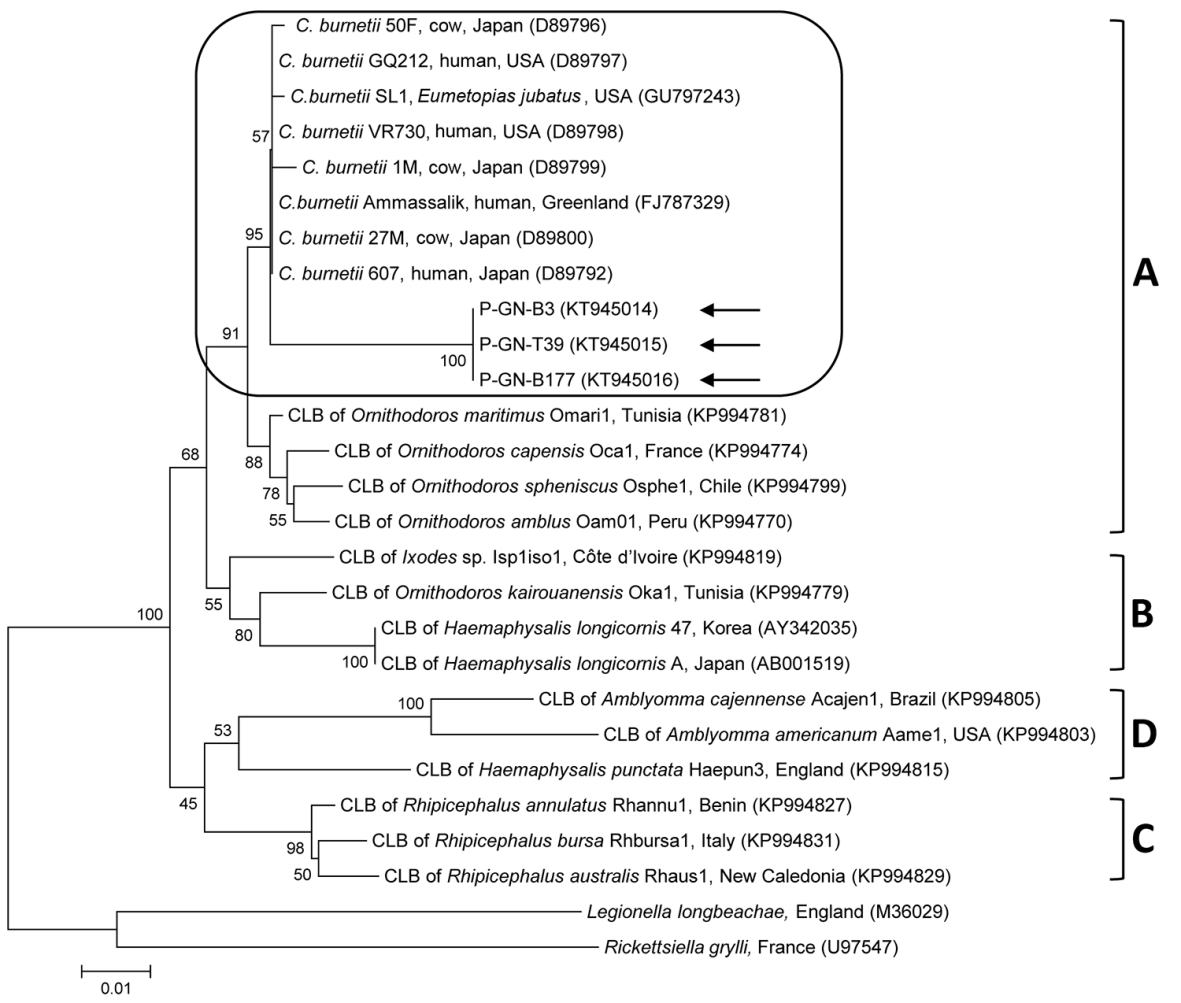
Phylogenetic tree constructed using the maximum-likelihood method from *Coxiella burnetii* 16S rRNA sequences. Arrows indicate *C. burnetii* sequences from study to detect and genotype *C. burnetii* in pigs in Gyeongsang Province, South Korea, 2014–2015. Rounded rectangle indicates *C. burnetii* group. The 4 *Coxiella* clades (A–D) are indicated at right. GenBank accession numbers for other sequences are shown in parentheses. Numbers on branches indicate bootstrap support (1,000 replicates). Scale bar represents the evolutionary distance between sequences. CLB, *Coxiella*-like bacteria.

Three (0.3%) pigs were positive for *C. burnetii* by PCR; all were breeding pigs and seronegative for *C. burnetii*. One positive sample was lung tissue from a pig that appeared to have respiratory signs; other respiratory pathogens were also detected in the sample. Acute *C. burnetii* infection with organ involvement was confirmed by PCR. However, the infection status of seropositive pigs cannot be determined on the basis of a single titer. *C. burnetii*–seronegative pigs can, however, shed the organism and, thus, might serve as a reservoir for transmission of the bacterium to humans. 16S rRNA gene sequences for the 3 *C. burnetii* PCR-positive samples (GenBank accession nos. KT945014–16; Figure 2) showed 100% identity with each other; nucleotide sequences showed high (96.6%–96.9%) identity with those of other *C. burnetii* strains. Phylogenetic analysis showed that the 3 isolates belong to clade A, clustering with previously published *C. burnetii* sequences (Figure 2).

## Conclusions

We found that 6.8%, 5.2%, and 0.3% of tested pig samples in Gyeongsang Province were positive for *C. burnetii* by ELISA, IFA, and PCR, respectively. These rates of seropositivity are relatively low compared with the rate found in a study in Uruguay, in which 18.4% (83/479) of the blood samples were seropositive by layer microagglutination (*7*). In that study, the innate susceptibility of pigs to *C. burnetii* was confirmed during a Q fever epidemic. Seropositivity in our study was, however, higher than that reported in blood tested by IFA in Japan (0/396 samples) (*8*) and by complement fixation in Bulgaria (0.05%; 1/1,809 samples) (*9*). 

In *C. burnetii*–positive animals, bacterial burden is highest in birth products. We did not test such tissues; however, the positivity rate in our study was similar to that (0/16) in a previous examination of pig placentas by real-time PCR in the Netherlands (*10*). In our study, the PCR-positive pig samples did not test positive by serologic methods.

Similar to results from a previous study (*11*), our results showed that IFA was less sensitive than ELISA at detecting *C. burnetii* in serum. However, serologic diagnosis of coxiellosis in animals is complicated. Animals can maintain seropositivity after acute infection has cleared, and they can seroconvert without shedding (*12*); thus, serologic methods are not useful for determining which animals currently pose a risk for transmission.

In our study, seroprevalence among breeding pigs was significantly high (p<0.05). In addition, only breeding pigs were positive for *C. burnetii* by PCR. Because of pregnancy stress, breeding pigs probably experienced a recrudescent infection, making them more likely to shed the organism. A study on the epidemiology of Q fever suggested that breeding pigs can cause infection in humans (*13*).

The genus *Coxiella* is divided into 4 highly divergent genetic clades (A–D); *C. burnetii* belongs to clade A (*14*). Phylogenetic analysis showed that the 3 *C. burnetii* isolates in our study were closely related to clade A strains from the United States, Japan, and Greenland, indicating a close epidemiologic link.

Although the number of *C. burnetii*–positive pigs was low in our study, a previous study identified contact with pigs as a risk factor for *C. burnetii* seropositivity in humans (*15*). Therefore, pigs may serve as potential reservoirs for *C. burnetii*. However, several questions remain unanswered regarding the epidemiology of *C. burnetii* infection in pigs and possible transmission to humans. Additional investigations of the infection prevalence in other animals are necessary to understand the epidemiology of *C. burnetii*.

Technical Appendix Methodology used in study of *Coxiella burnetii* in pigs in South Korea.
