# Supplementary material for: Detection and Genotyping of Coxiella burnetii in Pigs, South Korea, 2014–2015
Source: Emerg Infect Dis. 2016 Dec;22(12):2192–5. doi: 10.3201/eid2212.161236 (PMC5189167; doi:10.3201/eid2212.161236)
Supplement: Technical Appendix — Methodology used in study of Coxiella burnetii in pigs in South Korea. [file 16-1236-Techapp-s1.pdf]

Article DOI: http://dx.doi.org/10.3201/eid2212.161236

Detection and Genotyping of *Coxiella burnetii* in Pigs, South Korea, 2014–2015

Technical Appendix

Simple Random Sampling

The sample size was determined using the following formula, with an expected disease prevalence of 50%, accepted absolute error of 5%, and confidence level of 99% in a simple random sampling design (*1*):

$$n = \frac{1.96^2 p_{exp}(1 - p_{exp})}{d^2}$$

where n = the required sample size, p_{exp} = expected prevalence, and d = desired absolute precision.

Molecular Detection of *Coxiella*

The AccuPower HotStart PCR Premix Kit (Bioneer, Daejeon, South Korea) was used for PCR amplification. Nested PCR (nPCR) was conducted to amplify the 16S rRNA of *C. burnetii* and *Coxiella*-like bacteria (CLB), and sequencing differentially identified *C. burnetii* from CLB (*2*). First-round PCR was performed with the primers Cox16SF1 (5′-CGTAGGAATCTACCTTRTAGWGG-3′) and Cox16SR2 (5′-GCCTACCCGCTTCTGGTACAATT-3′), which produced amplicons of 1,321–1,429 bp. Then, nPCR was performed using the primers Cox16SF2 (5′-TGAGAACTAGCTGTTGGRRAGT-3′) and Cox16SR2, which produced amplicons of 624–627 bp. Samples yielding amplicons of the expected size were bi-directionally sequenced using the primers Cox16SF1 and Cox16SR1 (5′-ACTYYCCAACAGCTAGTTCTCA-3′), which produced amplicons of 719–826 bp. All PCR amplifications were performed using a Mastercycler Pro (Eppendorf, Hamburg, Germany) with pre-denaturation at 93°C for 3 min followed by 30 cycles of denaturation at 93°C for 30 s, annealing at 56°C for 30 s, and polymerization at 72°C for 1 min, and a post-polymerization step

at 72°C for 5 min. PCR products (10 μL) from the second amplification, along with a 100-bp DNA ladder (Intron Biotechnology, Seongnam, South Korea), were electrophoresed on 1.5% agarose gels at 100 V for 30 min and then visualized by ultraviolettransillumination after ethidium bromide staining.

Phylogenetic Analysis

The results were analyzed using the multiple sequence alignment program CLUSTAL Omega (ver.1.2.1; http://www.ebi.ac.uk/Tools/msa/clustalo/), and a homology comparison of the *Coxiella* 16S rRNA gene was performed. Alignments were edited using BioEdit (ver.7.2.5; http://www.mbio.ncsu.edu/bioedit/bioedit.html). Phylogenetic analysis was performed using MEGA (ver.6.0; http://megasoftware.net/) following the maximum likelihood method. The stability of the trees obtained was estimated by bootstrap analysis with 1,000 replicates.

Statistical Analysis

The chi-square test was used to analyze significant differences between the groups, where p values <0.05 were considered statistically significant. The analytical software package GraphPad Prism (ver.5.04; http://www.graphpad.com/scientific-software/prism/) was used for statistical analysis. A 95% CI was determined for all estimates. To compare the diagnosis methods, inter-assay agreement between ELISA and IFA in detecting *C. burnetii* was measured using Cohen's kappa coefficient, calculated with GraphPad QuickCalcs (http://graphpad.com/quickcalcs/kappa1.cfm). Kappa values ≤0.20 represented poor agreement, values between 0.21 and 0.40 represented fair agreement, those between 0.41 and 0.60 represented moderate agreement, and those between 0.61 and 0.80 represented good agreement, whereas values between 0.81 and 1.00 represented very good agreement. The Cohen's kappa coefficient must be interpreted with caution, as this value only indicates agreement, without suggesting the validity of the two tests (*1*).

GenBank Accession Numbers for Nucleotide Sequence of *Coxiella burnetii*

P-GN-B3	KT945014
P-GN-T39	KT945015

P-GN-B177 KT945016

References

1. Thrusfield M. Veterinary epidemiology, 3rd ed. Oxford, UK: Wiley Blackwell; 2005. p. 228–330.

2. Duron O, Noël V, McCoy KD, Bonazzi M, Sidi-Boumedine K, Morel O, et al. The recent evolution of a maternally-inherited endosymbiont of ticks led to the emergence of the Q fever pathogen, *Coxiella burnetii*. PLoS Pathog. 2015;11:e1004892. PubMed http://dx.doi.org/10.1371/journal.ppat.1004892
